# Supplementary material for: The causal relationship between the human gut microbiota and pyogenic arthritis: a Mendelian randomization study
Source: Front Cell Infect Microbiol. 2024 Nov 26;14:1452480. doi: 10.3389/fcimb.2024.1452480 (PMC11629706; doi:10.3389/fcimb.2024.1452480)
Supplement: Supplementary file 1 [file DataSheet1.zip › mendelian test/ebi-a-GCST90027643.csv_forest.pdf]

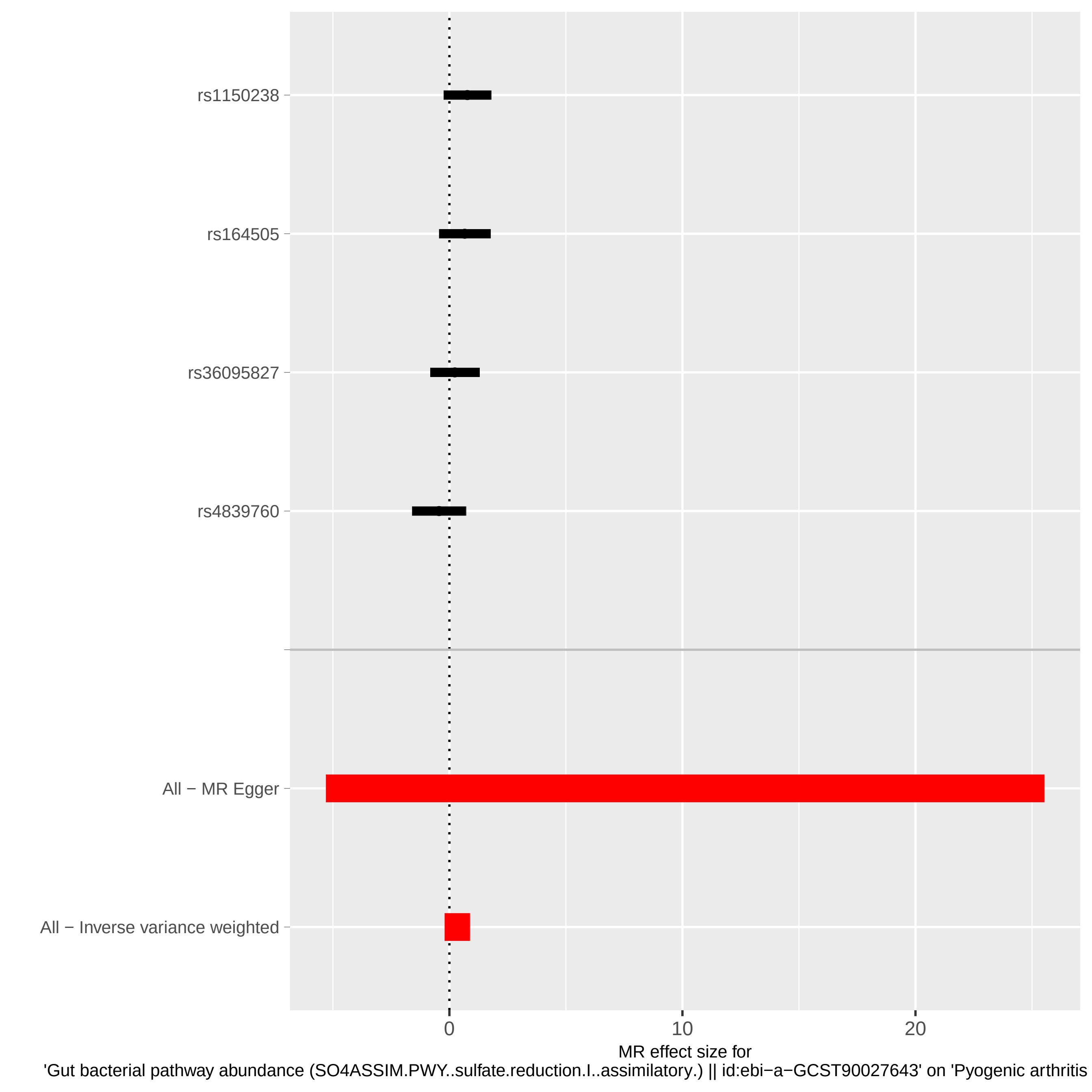

rs1150238
rs164505
rs36095827
rs4839760
All − MR Egger
All − Inverse variance weighted
0
10
20
MR effect size for
'Gut bacterial pathway abundance (SO4ASSIM.PWY..sulfate.reduction.I..assimilatory.) || id:ebi−a−GCST90027643' on 'Pyogenic arthritis
